# A survey of orphan enzyme activities

**DOI:** 10.1186/1471-2105-8-244

**Published:** 2007-07-10

**Authors:** Yannick Pouliot, Peter D Karp

**Affiliations:** 1 Bioinformatics Research Group, Artificial Intelligence Center, SRI International, 333 Ravenswood Ave, Menlo Park, California, 94025-3493, USA; 2 Lane Medical Library and Knowledge Management Center, Information Resources and Technology, Stanford University Medical Center, 300 Pasteur Drive. Stanford, CA 94305-5123, USA

## Abstract

**Background:**

Using computational database searches, we have demonstrated previously that no gene sequences could be found for at least 36% of enzyme activities that have been assigned an Enzyme Commission number. Here we present a follow-up literature-based survey involving a statistically significant sample of such "orphan" activities. The survey was intended to determine whether sequences for these enzyme activities are truly unknown, or whether these sequences are absent from the public sequence databases but can be found in the literature.

**Results:**

We demonstrate that for ~80% of sampled orphans, the absence of sequence data is bona fide. Our analyses further substantiate the notion that many of these enzyme activities play biologically important roles.

**Conclusion:**

This survey points toward significant scientific cost of having such a large fraction of characterized enzyme activities disconnected from sequence data. It also suggests that a larger effort, beginning with a comprehensive survey of all putative orphan activities, would resolve nearly 300 artifactual orphans and reconnect a wealth of enzyme research with modern genomics. For these reasons, we propose that a systematic effort to identify the cognate genes of orphan enzymes be undertaken.

## Background

After a decade of comprehensive genomic sequencing, more than 500 genomes have been sequenced to completion, mostly prokaryotes. The prodigious rate of new sequence annotation is highlighted by the fact that there were just over 300 genomes available when this study was carried out in late 2004. However, the fraction of genes for which no function can be predicted remains high (30%–50%). In response, proposals have been put forth for the bioinformatics analysis of bacterial genomes to identify genes with high likelihood of scoring true in confirmatory laboratory assays of their respective function [1,2]. This would increase the field's pool of experimentally characterized proteins, with concomitant increases in the accuracy and coverage of genome annotation. We believe the return on investment of this approach would be particularly high when addressing the problem of orphan activities, that is, enzymatic activities for which no sequence information is available [3,4].

**Figure 1:**
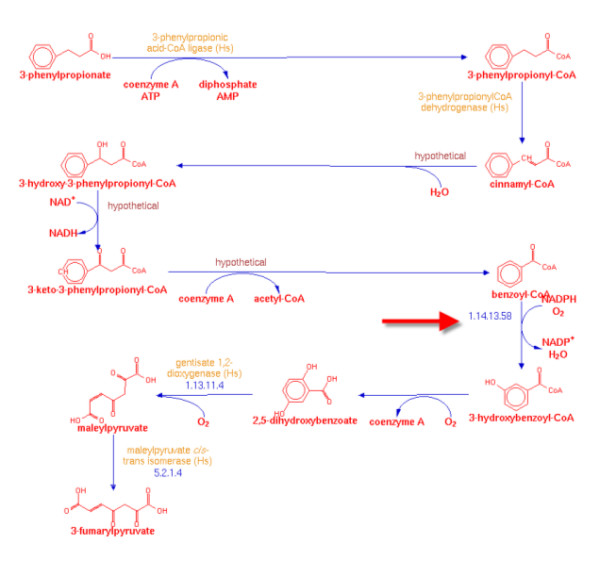
Example of a metabolic pathway involving a validated orphan.

Decades of detailed enzymology have created a wealth of knowledge about enzymes and their activities. However, crucial aspects of these enzymes are absent from bioinformatics databases with surprising frequency. For example, recent computational analyses of sequence databases demonstrate that at least 36% of enzyme activities that have been assigned an Enzyme Commission (EC) number [5] appear to be devoid of a gene or protein sequence [3]. Since then, similar analyses have been published, with similar results [4,6,7]. The existence of such a large fraction of orphan activities is surprising, given that many of these enzymes have been described decades ago and are often involved in basic cellular functions. Several examples exist of the recent identification of genes involved in important enzymatic functions (reviewed in [1,2]). Indeed, in our study 44 orphans were found to be present in one or more primary metabolic pathways in a variety of species (described below). Details of many of the orphan enzymes uncovered during this survey point to multiple and significant consequences for the lack of sequence information in areas such as genome annotation, computational pathway prediction, and metabolic engineering. For these reasons, the orphan problem and related issues were highlighted in a recent report of the American Society for Microbiology [8]. In view of the biological richness associated with orphan enzymatic activities (Figure 1, Table 1), we have taken the first steps in creating the foundations of an Enzyme Genomics Initiative [3].

**Table 1:** Biological significance of selected validated orphans. The extent and significance of published research associated with a selection of validated orphans is detailed

**EC No**.	**Activity**	**Year first published**	**No. PubMed Publications Involving Orphan**	**Significance**
1.1.1.43	Phosphogluconate 2-dehydrogenase	1961	2417	Positive reports of evaluation as a drug target against Trypanosome; trypanocidal activity has been reported; involved in 2-dehydro-D-gluconate degradation pathway
*2.3.1.23*	*1-acylglycerophosphocholine O-acyltransferase*	*1967*	*256*	*Activity is present in lower eukaryotes, plants, and multiple mammalian tissues*
5.1.3.17	Heparosan-*N-*sulfate-glucuronate 5-epimerase	1979	16	Involved in the biosynthesis of heparan sulfate, which binds proteins to modulate signaling events in embryogenesis. Mouse gene knock-out results in late lethal phenotype. Correction added in proof: Thanks to a comment by Dr. K. Robison and research by Dr. A. Shearer, we have found that 5.1.3.17 is an artifactual orphan rather than a validated orphan. Genes for this enzyme have been identified in cow and mouse (J Biol Chem 272:28158 1997; J Biol Chem 276:20069 2001).
2.3.1.105	Alkylglycerophosphate 2-O-acetyltransferase	1986	9	Involved in platelet activating factor biosynthesis; possible involvement in ischemia
3.1.3.59	Alkylacetylglycerophosphatase	1986	9	Involved in platelet activation factor biosynthesis
2.7.1.106	Glucose-1,6-bisphosphate synthase	1975	9	Present in several mammalian tissues. Involved in glucose metabolism
1.2.1.23	2-oxoaldehyde dehydrogenase (NAD+)	1967	9	Involved in the development of diabetic complications
1.14.11.6	Thymine dioxygenase	1972	9	Present in both lower and higher eukaryotes
1.1.1.16	Galactitol 2-dehydrogenase	1956	5	Insulin dysregulation
2.3.1.14	Glutamine N-phenylacetyltransferase	1957	4	Investigated as a predictor of carotid endarterectomy in middle-aged individuals
1.2.1.25	2-oxoisovalerate dehydrogenase (acylating)	1969	4	Present in prokaryotes and eukaryotes. In the latter, participates in primary metabolism pathway for valine degradation

E.C. 2.3.1.23 is listed in italics because it was cloned and sequenced in 2006, after the completion of this study

**Figure 2:**
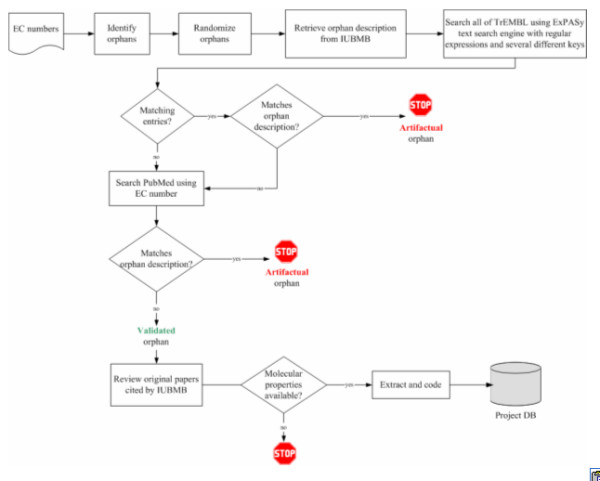
Literature survey process.

Here we describe a literature-based survey of presumed orphans intended to further validate and characterize these activities (Figure 2). The confidence of the results of this survey was designed to be within a 5% error margin relative to the universe of orphan activities, based on a randomly selected subset of orphan activities from the Nomenclature Committee of the IUBMB (NC-IUBMB). We have also assessed the practicability of identifying the genes associated with these orphans. As a consequence, the survey captures data from the literature that should facilitate the identification of cognate genes for the orphan activities evaluated. Here, we define the cognate gene for an activity as a gene that has been shown to code for an enzyme that carries out that activity.

The survey confirmed that ~80% of the sampled orphans do not have sequence information associated with them. Consequently, this lack represents a true information deficit. Weaknesses in database integration and a lack of information capture from the literature to databases appear to be largely responsible for most of the artifactual orphans making up the other 20%. Given the importance of these enzymatic activities, we propose that the public sequence databases assign high priority to correcting database entries for artifactual orphans. We further propose that a systematic effort be undertaken to sequence the genes of validated orphans, as this survey demonstrates that primary literature data and database analyses combined with current proteomics and genomic technologies should be adequate to enable the rapid identification of many of these genes.

## Results

Most orphan enzymatic activities are *bona fide *(Table 2). Our survey found that more than 80% of orphans are not due to artifacts such as missing database annotations (primarily failure to capture information from the literature), or lack of database cross-referencing, such as the availability of a sequence in one database not being reflected in a second database. Specifically, a total of 187 orphans out of 228 surveyed activities were validated in at least one of 287 species (species are listed in Table 3 and Table 4, the list of validated orphans is in Table 5). A majority of orphans (54.36%) occurred in Eukaryotes, followed by Eubacteria (39.37%) (Table 6). Within the Eubacteria, genus *Pseudomonas *was significantly overrepresented (35%) (Table 7). While a systematic determination of the species spectrum of orphan activities was not performed here, we did notice several cases of an orphan activity reported in more than one species, as well as one case of an orphan activity occurring in species from different domains.

**Table 2:** Summary of survey results

	**Number**	**Proportion**
	
Total number of putative orphans	1,356	
Number required to achieve 95% significance	180	13.3%
Number orphans evaluated	228	16.8%
		
Out of 228 orphans:		
Number of artifactual orphans	41	18.0%
Number of valid orphans	187	82.0%
Max. number of salvageable orphans (all rankings)	57	25.0%
		
Out of 57 salvageable orphans:	**Number**	**Proportion**
	
Excellent	9	15.8%
Good	23	40.4%
Marginal	9	15.8%
Poor	16	28.1%
Bacterial salvageable orphans	26	45.6%
Eukaryotic salvageable orphans	31	54.4%

The survey was designed to achieve a maximum sampling error of 5%, 19 times out of 20. This corresponds to a minimum sample size of ~80 orphans. A total of 228 orphans were in fact surveyed. In a number of cases more than one instance of an orphan activity was evaluated because the activity was reported in more than one species. Consequently, 286 instances were evaluated.

**Table 3:** Species distribution of Eubacterial validated orphans

Species	No. of Orphans	Species	No. of Orphans
Acinetobacter NCIB 9871	1	Pasteurella tuberculosis	2
Actinoplanes missouriensis	1	Pedobacter heparinus	1
Aerom onas sp.	1	Propionibacterium pentosaceum	1
Alcaligenes eutrophus	1	Proteus mirabilis	1
Alcaligenes faecalis	1	Pseudomonas (species undefined)	2
Arthrobacter GJM -1	1	Pseudomonas fluorescens	3
Arthrobacter oxydans	1	Pseudomonas graveolens	1
Arthrobacter sp.	2	Pseudomonas MS	1
Azotobacter vinelandii	1	Pseudomonas MSU-1	1
Bacillus subtilis	1	Pseudomonas P-2	2
Cellulom onas sp.	1	Pseudomonas putida	7
Clostridium cylindrosporum	1	Pseudomonas putida P2	1
Clostridium kluyveri	2	Pseudomonas saccharophilia	2
Clostridium pasteurianum	1	Pseudomonas sp.	4
Clostridium SB4	1	Pseudomonas sp. P-501	1
Clostridium sporogenes	2	Pseudomonas syringae GG	1
Corynebacterium cyclohexanicum	1	Pseudomonas testosteroni	1
Escherichia coli	8	Rhodococcus	1
Flavobacterium	1	Rhodopseudomonas sphaeroides	1
Flavobacterium sp.	1	Salmonella typhimurium	1
Klebsiella aerogenes	1	Streptococcus faecalis	1
Micrococcus denitrificans	1	Streptococcus mutans	1
Microorganism	2	Streptomyces virginiae	1
Mycobacterium tuberculosis	1	Thiobacillus thioparus	1
Nocardia (species undefined)	1	Unknown	2

The total number of orphans is greater than the number of activities because a given activity may be present in more than one species. The exact species of some orphans can be unclear or unstated, in which case these are classified under a generic term ("species undefined", "unknown", etc). The total number of orphans is greater than the number of activities because a given activity may be present in more than one species. The exact species of some orphans can be unclear or unstated, in which case these are classified under a generic term ("species undefined", "unknown", etc).

**Table 4:** Species distribution of Eukaryotic validated orphans

**Species**	**No. of Orphans**	**Organism**	**No. of Orphans**
Acrocylindrium sp.	1	Nectria haem atococca/Fusarium solani f.sp. Phaseoli	1
Arachis hypogaea	2	Neurospora (subspecies undefined)	1
ASparagus officinalis	1	Neurospora crassa	1
Aspergillus niger	2	Ochromonas malhamensis	1
Avena coleoptiles	1	Ovis aries	2
Bauhenia monandra	1	Pea sativum var. Alaska	1
Bostaurus	3	Penicillium atrovenetum	1
Capra hircus	1	Penicillium chrysogenum	1
Catharanthus roseus	1	Penicillium patulum	1
Cavia porcellus	3	Phaseolus aureus	3
Chlorella	1	Phaseolus radiatus	1
Chrysosplenium americanum	1	Pisum sativum (variety unspecified)	1
Cichorium endivia	1	Pycnoporus coccineus	1
Citrus (subspecies undefined)	1	Raphanus sativus	1
Corydalis cava	1	Rat (subspecies undefined)	18
Cucurbita maxima	1	Rat Sprague-Dawley	5
Daucus carota	1	Rhodotorula glutinis	2
Entamoeba histolytica	1	Saccharomyces cerevisiae	5
Euglena gracilis	1	Saccharum officinarum	1
Flaveria spp.	1	Secale cereale	1
Fundulus heteroclitus	1	Sesamum indicum	1
Gallus gallus	1	Several	1
Homo sapiens	5	Sorghum bicolor	2
Hordeum (species undefined)	1	Spinacia	1
Hordeum vulgare subsp. Vulgare	2	Spinacia oleracea	1
Lasallia pustulata	1	Sus scrofa	7
Lilium longiflorum	1	Tecoma stans	1
Lupinus albus	1	Thea sinensis	1
Lycopersicon esculentum	2	Trypanosoma brucei brucei	1
Macaca mulatta	1	Tulipa cv. Apeldoorn	1
Mentha piperita	1	Unknown	1
Mesocricetus auratus	1	Yeast (species undefined)	4
Mold	1	Zea mays	2
Mouse (species undefined)	3		

The total number of orphans is greater than the number of activities because a given activity may be present in more than one species. The exact species of some orphans can be unclear or unstated, in which case these are classified under a generic term ("mold", "mouse", "unknown", etc). The total number of orphans is greater than the number of activities because a given activity may be present in more than one species. The exact species of some orphans can be unclear or unstated, in which case these are classified under a generic term ("mold", "mouse", "unknown", etc).

**Table 5:** Validated orphan activities

**EC No**.	**Ranking**	**EC No**.	**Ranking**	**EC No**.	**Ranking**
1.1.1.13	difficult	1.14.99.24	difficult	3.1.3.47	good
1.1.1.16	difficult	1.21.3.2	difficult	3.1.3.59	difficult
1.1.1.43	difficult	1.97.1.3	difficult	3.1.3.72	difficult
1.1.1.54	difficult	2.1.1.112	difficult	3.1.4.43	difficult
1.1.1.84	excellent	2.1.1.137	artifact	3.1.6.17	good
1.1.1.92	marginal	2.1.1.141	artifact	3.1.8.2	artifact
1.1.1.101	difficult	2.1.1.143	artifact	3.2.1.56	difficult
1.1.1.144	difficult	2.1.1.147	difficult	3.2.1.77	difficult
1.1.1.146	artifact	2.1.1.84	difficult	3.2.1.100	good
1.1.1.163	artifact	2.1.1.99	difficult	3.2.1.112	excellent
1.1.1.172	good	2.1.2.4	difficult	3.2.1.115	difficult
1.1.1.196	good	2.3.1.14	difficult	3.2.1.128	excellent
1.1.1.208	poor	2.3.1.23	difficult	3.2.1.136	difficult
1.1.1.226	excellent	2.3.1.24	artifact	3.2.1.137	poor
1.1.1.245	artifact	2.3.1.33	difficult	3.2.2.10	difficult
1.1.1.258	artifact	2.3.1.49	difficult	3.4.11.16	good
1.1.1.265	excellent	2.3.1.68	marginal	3.4.13.7	difficult
1.1.2.5	artifact	2.3.1.96	difficult	3.4.17.16	good
1.1.3.23	difficult	2.3.1.98	good	3.4.21.103	artifact
1.17.1.1	difficult	2.3.1.102	artifact	3.4.22.44	artifact
1.17.99.2	artifact	2.3.1.103	poor	3.4.22.46	artifact
1.2.1.18	artifact	2.3.1.105	poor	3.4.23.28	difficult
1.2.1.20	difficult	2.3.1.114	poor	3.4.23.30	artifact
1.2.1.23	poor	2.3.1.133	marginal	3.4.24.54	artifact
1.2.1.25	difficult	2.3.1.140	difficult	3.5.1.30	good
1.2.1.32	artifact	2.3.1.161	artifact	3.5.1.33	artifact
1.2.1.33	difficult	2.3.2.3	artifact	3.5.1.39	poor
1.2.1.52	difficult	2.3.2.7	difficult	3.5.1.58	excellent
1.2.1.54	good	2.3.3.3	difficult	3.5.1.62	artifact
1.2.1.63	marginal	2.4.1.23	poor	3.5.1.67	difficult
1.2.1.64	artifact	2.4.1.29	difficult	3.5.1.71	poor
1.2.3.6	difficult	2.4.1.41	valid	3.5.1.79	artifact
1.2.3.7	poor	2.4.1.43	difficult	3.5.2.13	poor
1.2.3.8	artifact	2.4.1.57	artifact	3.5.2.16	artifact
1.3.1.4	difficult	2.4.1.66	artifact	3.5.3.2	good
1.3.1.5	difficult	2.4.1.73	artifact	3.5.5.2	difficult
1.3.1.6	artifact	2.4.1.97	difficult	3.6.1.18	excellent
1.3.1.11	difficult	2.4.1.110	poor	3.6.1.2	artifact
1.3.1.37	difficult	2.4.1.125	difficult	3.6.1.52	artifact
1.3.7.1	difficult	2.4.1.126	valid	3.6.3.17	artifact
1.3.99.15	artifact	2.4.1.153	poor	3.6.3.24	artifact
1.3.99.21	artifact	2.4.1.167	difficult	3.6.3.28	artifact
1.4.1.11	good	2.4.1.176	difficult	3.6.4.4	artifact
1.4.1.17	good	2.4.1.180	excellent	4.1.1.24	difficult
1.4.99.4	marginal	2.4.1.184	difficult	4.1.1.52	difficult
1.4.99.5	artifact	2.4.1.215	artifact	4.1.1.56	difficult
1.5.1.21	good	2.4.2.35	difficult	4.1.1.75	difficult
1.5.99.11	artifact	2.5.1.4	difficult	4.1.2.23	difficult
1.6.5.7	artifact	2.5.1.42	difficult	4.1.2.28	difficult
1.7.3.1	difficult	2.6.1.22	difficult	4.1.2.35	difficult
1.7.3.5	valid	2.6.1.27	poor	4.1.3.35	difficult
1.8.1.5	artifact	2.6.1.32	difficult	4.2.1.5	difficult
1.10.1.1	difficult	2.6.1.33	poor	4.2.1.43	good
1.10.3.4	difficult	2.6.1.75	good	4.2.1.62	good
1.12.98.2	artifact	2.7.1.43	difficult	4.2.1.77	difficult
1.13.11.14	difficult	2.7.1.54	difficult	4.2.1.81	difficult
1.13.11.24	artifact	2.7.1.64	difficult	4.2.1.93	difficult
1.13.11.25	artifact	2.7.1.77	difficult	4.2.1.97	marginal
1.13.11.35	difficult	2.7.1.106	difficult	4.2.1.101	artifact
1.13.12.9	good	2.7.1.131	poor	4.2.2.14	artifact
1.14.11.10	difficult	2.7.1.134	difficult	4.2.3.19	artifact
1.14.11.6	poor	2.7.1.142	difficult	4.2.99.19	artifact
1.14.13.10	marginal	2.7.4.20	difficult	4.3.1.10	difficult
1.14.13.23	good	2.7.7.44	difficult	4.3.1.20	difficult
1.14.13.24	artifact	2.7.7.51	difficult	4.5.1.4	difficult
1.14.13.42	difficult	2.7.8.10	difficult	5.1.1.6	difficult
1.14.13.51	difficult	2.7.8.22	difficult	5.1.1.9	marginal
1.14.13.58	excellent	2.8.1.3	excellent	5.1.3.17	difficult
1.14.13.60	difficult	2.8.2.28	difficult	5.2.1.10	difficult
1.14.13.72	difficult	3.1.1.36	difficult	5.2.1.11	difficult
1.14.13.73	artifact	3.1.1.39	difficult	5.4.3.5	artifact
1.14.15.2	difficult	3.1.1.40	poor	5.5.1.11	good
1.14.16.5	good	3.1.1.78	artifact	5.5.1.12	artifact
1.14.99.18	artifact	3.1.2.11	difficult	5.5.1.3	difficult
1.14.99.22	artifact	3.1.3.14	difficult	6.3.1.6	difficult
		3.1.3.38	poor	6.3.4.8	difficult

All 228 orphans reviewed in this study are listed. The salvageability of an orphan is ranked "difficult" when factors such as unclear species of origin, lack of molecular descriptors, or lack of comprehensive genome sequence hinder cloning of the cognate gene. Note that such rankings do not take into account the availability of molecular descriptors which enable the identification of a candidate gene in one species, and, through orthology, the identification of a candidate gene in a second species for which these descriptors are not available.

**Table 6:** Domain distribution of validated orphans

**Domain**	**No. Species**	**Proportion**
Eukaryota	156	54.36%
Eubacteria	113	39.37%
Unknown	15	5.23%
Viruses	2	0.70%
Archaea	1	0.35%

Orphans with "Unknown" listed for their domain tend to be microbes that were insufficiently characterized to place them in either the Eubacteria or Archaea domains.

**Table 7:** Top four most represented Eubacteria

**Genus**	**No. instances of orphans**	**Fraction of all Eubacteria**
*Pseudomonas*	27	35.06%
*Escherichia*	8	10.39%
*Clostridium*	7	9.09%
*Arthrobacter*	4	5.19%

Because the eventual isolation of the cognate genes of these activities is greatly facilitated by comprehensive genome sequencing, we determined for what fraction of all validated orphans a full genome sequence is available (Table 8). 43% of Eubacterial species in which orphans occurred were found to have such sequences, available either presently or due shortly. This figure rises to 83% when including the genomes of related species, on the assumption that they might be sufficiently closely related to permit the identification of the cognate gene. For example, at the time of this study the completed genome sequence of *Pseudomonas fluorescens *was not available, but those of three other *Pseudomonas *species were.

**Table 8:** Availability of completely sequenced genomes for Eubacterial validated orphans

	**Complete genome sequence**		**Ongoing genome sequencing**	
	**Count**	**Proportion**	**Count**	**Proportion**
	
Same species	23	31.9%	9	18.4%
Same genus, related species	12	16.7%	16	32.6%

The number of available comprehensive genome sequences for validated Eubacterial orphans was tallied. Cases where the genome sequence of a species does not exist but where the sequence of a related species from the same genus is available are also listed, as are ongoing comprehensive genomic sequencing projects for genomes not currently available.

**Figure 3:**
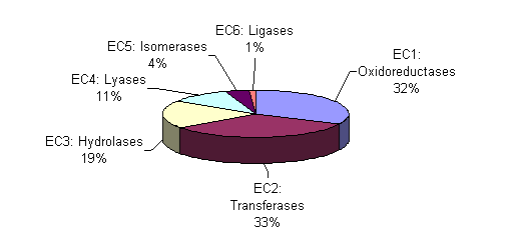
**Distribution of enzymatic activities in validated orphans**. The percentage of validated orphan activities belonging to each EC class is shown.

Oxidoreductases (EC1) and transferases (EC2) were the most frequently represented classes of enzymatic activity for validated orphans (Figure 3). On a per capita basis, oxidoreductases and transferases were overrepresented by ~20%, whereas hydrolases and ligases were underrepresented by 35% and 64%, respectively.

**Figure 4:**
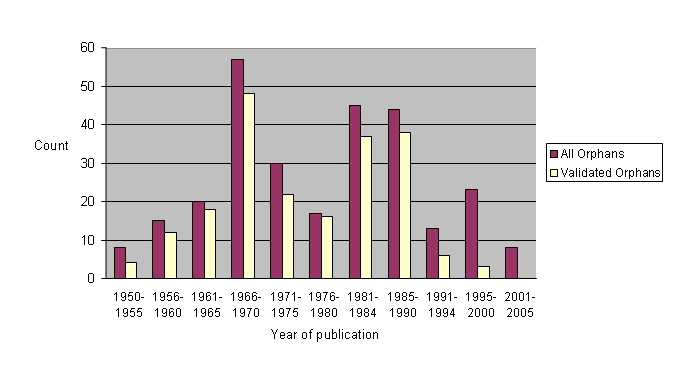
**Publication year of original publications describing orphan activities**. The publication date associated with the original source articles of all instances of orphans surveyed here is plotted (286 instances of orphans, corresponding to 228 activities), based upon the IUBMB record. In a number of cases more than one instance of an orphan activity was evaluated because the activity was reported in more than one species.

The original publication date for all orphans was broadly distributed around a mean of 1977 (Figure 4), compared to a mean of 1975 for validated orphans.

### Causes of artifacts

A comprehensive list of artifactual orphans and the inferred nature of the artifact is available [9]. Although this study was not designed to determine conclusively the causes of artifactuality, incompleteness in database entries appears to be the predominant cause of the artifacts identified here. For example, the DNA sequence associated with reaction 3.5.1.79 is available in the EMBL database, however, the UniProt entry for this enzyme does not list any protein sequence (Table 9). Other representative artifactual orphans are listed in Table 9, along with a description of the cause of the artifact. In a small fraction of cases a clear determination of the species in which the activity was characterized could not be made.

**Table 9:** Example artifactual orphans

**EC No**.	**Enzyme Name**	**Original Species**	**Year**	**Swiss-Prot/TrEMBL Acc. No**.	**Cause of artifact**	**Significance of error; importance of orphan activity**
3.4.21.103	Physarolisin (a proteinase)	*Physarum flavicomum*	1982	Q8MZS4	IUBMB entry lists a 2003 paper describing a gene coding for a protein with this activity [28]. Sequence is in Swiss-Prot but ENZYME does not reference this sequence.	Lack of database cross-referencing presumably involving the long interval between the initial characterization of the activity and the cloning of the gene.
3.5.2.16	Maleimide hydrolase	*Blastobacter sp. A17p-4*	1997	Q93T25	ENZYME and IUBMB entries are not referencing a Swiss-Prot entry from a 2002 paper describing the cloning of gene coding for this [29].	Lack of database cross-referencing is not restricted to older orphans.
3.5.1.79	Phthalyl amidase	*Xanthobacter agilis*	1995	N/A	The sequence, listed in a patent associated with a 1996 paper by [30] in *Journal of Molecular Catalysis B: Enzymatic *are available from Entrez, but not from TrEMBL. The paper itself is not available from PubMed.	Note: though the protein sequence is not available from the UniProt database, the DNA sequence is present in the EMBL database.
3.1.8.2	Diisopropyl-fluoro-phosphatase	*Alteromonas sp*.	1954	Q44238	ENZYME and IUBMB entries are not referencing a Swiss-Prot entry associated with a 1996 paper describing the cloning of a gene coding for an enzyme with this activity [31].	This enzymatic activity detoxifies nerve gas. The gene is part of a widespread gene family with otherwise unknown function, with members in *Homo sapiens*.

### Extent of salvageability

**Figure 5:**
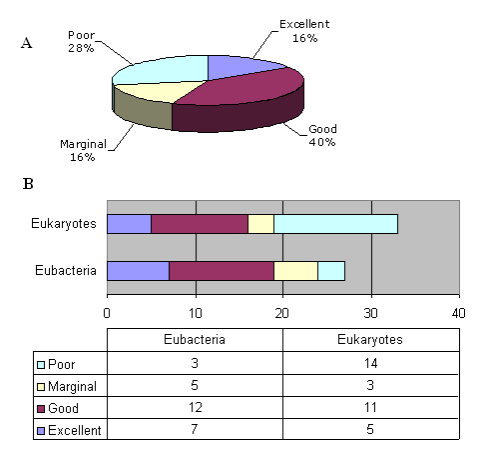
**Salvageability ranking of validated orphans**. The suitability of validated orphans for eventual cloning of at least one cognate gene was evaluated according to the ranking system described in the text. Out of 228 orphans, 57 were judged to be salvageable. A: Overall salvageability ranking (percentage out of 57); B: Domain distribution of salvageable orphans (number of orphans). Note that the total is greater than 57 because some orphans have different evaluations in the different species in which they have been reported. One orphan is also shared between Eubacteria and Eukaryotes.

Validated orphans were analyzed to determine whether sufficient information is available from their published characterization that, when combined with other factors, could enable the rapid identification of at least one cognate gene. Overall, we determined that 57 validated orphans (25% of total) might be salvageable (Figure 5A; Table 10), distributed approximately equally across eukaryotes and bacteria. Far more bacterial orphans were judged to have "excellent" or "good" salvageability as compared with eukaryotic orphans: 70% (7+12 out of 27) vs. 48% (5+11 out of 33), respectively (Figure 5B). This discrepancy is primarily due to factors such as the much greater difficulty for purifying an activity from higher eukaryotes, the difficulty of obtaining enough starting protein from lower eukaryotes such as multicellular fungi, and the absence of a comprehensive genome sequence from species such as *Bos Taurus and Sus scrofa*.

**Table 10:** Example artifactual orphans that are  salvageable

**EC No**.	**Ranking**	**EC No**.	**Ranking**
1.1.1.226	excellent	1.4.1.11	Good
1.1.1.265	excellent	1.4.1.17	Good
1.14.13.58	excellent	1.5.1.21	Good
2.4.1.180	excellent	2.3.1.98	Good
2.8.1.3	excellent	2.6.1.58	Good
3.2.1.112	excellent	2.6.1.75	Good
3.2.1.128	excellent	3.1.3.47	Good
3.5.1.58	excellent	3.1.6.17	Good
3.6.1.18	excellent	3.2.1.100	Good
1.1.1.172	good	3.4.17.16	Good
1.1.1.196	good	3.5.1.30	Good
1.13.12.9	good	3.5.3.2	Good
1.14.13.23	good	4.2.1.43	Good
1.14.16.5	good	4.2.1.62	Good
1.2.1.54	good	5.5.1.11	Good

Validated orphans with a salvageability ranking of "good" or better are listed.

**Figure 6:**
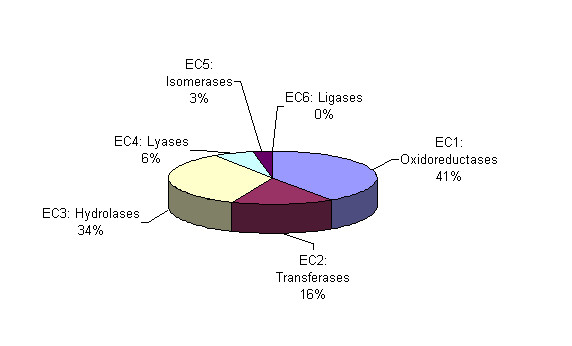
Distribution of enzymatic activities for salvageable orphans ranked "good" and "excellent".

Overall, more than half of the salvageable orphans ranked "good" or "excellent", with oxidoreductases (EC1) and hydrolases (EC2) being overrepresented in that set. All other enzymatic classes were significantly underrepresented (Figure 6).

## Discussion

This survey demonstrates that ~80% of orphan enzymatic activities are *bona fide; *therefore, we conclude that of the 1,356 putative orphans extant at the time of this study, more than 1,000 are highly likely to constitute true information deficits since their lack of sequence information is not the result of a database error.

The absence of DNA or protein sequences encoding such well-characterized enzymatic activities is particularly consequential because these activities were often identified decades ago, and many have been the focus of significant research activity (Table 1). Without the cognate sequences for these activities, the quality of annotation of all sequenced genomes in terms of both coverage (fraction of genes that can be recognized) and accuracy (fraction of predicted gene functions that are correct) is diminished. Many of these activities may go for years without being sequenced – for example, 1-acylglycerophosphocholine O-acyltransferase (Table 1) was finally purified and sequenced nearly forty years after it was first characterized [10]. Perhaps more troubling is the unknown pool of "false positive" annotations. Phosphogluconate 2-dehydrogenase (Table 1), an orphan at the time of this analysis, has since been assigned to a sequence in the human genome with no experimental evidence linking it to that or any homologous sequence, but apparently instead on the basis of the gene in question already being assigned a similar activity. This kind of "hidden orphan" would have been missed by most orphan analyses, and can be expected to propagate a potentially incorrect assignment to other genomes in the future. Computational metabolic pathway prediction [11] and metabolic engineering also depend on sequence information and are thus similarly compromised.

Conversely, ~20% of orphans surveyed were observed to be artifacts, such that ~270 orphans out of 1,356 putative orphans examined should be resolvable entirely via literature research and database cleanup. As a result of this process as it was carried out on our sampling of orphans, we have reported 11 artifactual orphan activities to public sequence repositories for correction (see Table 8 for examples).

In addition to validating orphans, the survey was useful in capturing information from the literature to assess their salvageability: more than half of validated orphans were found to be salvageable (Figure 5). Examples of salvageable orphan activities with the traits that make them salvageable are listed in Table 11.

**Table 11:** Selected salvageable orphans

**EC No**.	**Ranking**	**Pathways**	**Activity**	**Species**	**Full Genome Sequence?**	**Ongoing Genomic Sequencing?**	**Mr (kDa)**	**pI (pH units)**
3.5.1.30	Good	None	5-amino-penta-namidase	*Pseudomonas putida *P2, *Pseudomonas fluorescens*	Yes (*P. putida*)*	Several *Pseudomonas *species	67	N/A
5.5.1.11	Good	None	Dichloro-muconate cyclo- isomerase	*Alcaligenes eutrophus *JMP 134 (*Ralstonia eutropha *JMP134)	N/A	Yes	40 ± 10	N/A
4.2.1.97	Marginal	None	Phaseollidin hydratase	*Fusarium solani f.sp. Phaseoli*	No	Different species (*Fusarium sporotrichioides)*	monomer 1: 47 monomer 2: 49	
2.3.1.103	Poor	None	Sinapoylglucose–sinapoylglucose O-sinapoyltransferase	*Raphanus sativus*	N/A	N/A	55	N/A

A selection of orphans with different salvageability rankings are listed. Pathway names are those used in the MetaCyc database. *: The genomes of several strains of *P. fluorescens *are in the final stages of assembly and are essentially fully sequenced. N/A: not available

As abundantly noted elsewhere, such database cleansing is essential to maximize the existing research investment and prevent the propagation of mistakes [12-14] (see Table 12 for examples of artifacts that have been resolved). This necessity has not eluded the field of enzymology [3,4,15,16], and the present survey demonstrates the usefulness of correlating biological databases and mining the literature to enhance the value of existing research and facilitate the identification of the remaining orphan-associated genes. Until recently, there were no general repositories of orphan activity data, although some species-specific databases and pages were maintained, such as EchoBase [17] and a web page listing unidentified *E. coli *enzymes maintained by the EcoCyc project [18]. Consequently, we updated the MetaCyc [19] database to identify reactions that have been analyzed by this survey, and annotated them and associated database objects with results such as the validity of their orphan status, links to their cognate protein in the case of artifacts, and the properties of the protein copurifying with the activity in the case of validated orphans. Recently, Lespinet and Labedan created ORENZA [20], a database dedicated to maintaining an up-to-date listing of all enzyme activities for which no sequences are available in major sequence databases [6]. We are contributing our updated orphan information to ORENZA as well. These data, captured in MetaCyc and ORENZA, should facilitate the work of enzymologists interested in identifying the cognate genes of orphan activities. For instance, the work of Melnick *et al*. [21] is an excellent example of the combined application of modern laboratory and bioinformatics techniques that would benefit from the data described here.

**Table 12:** Example of artifactual orphans resolved by this survey

**EC No**.	**Enzyme Name**	**Species**	**TrEMBL/Swiss-Prot Accession No**.
1.1.1.163	Cyclopentanol dehydrogenase	*Comamonas sp*.	Q8GAV9
1.13.11.24	Quercetin 2,3-dioxygenase	*Bacillus subtilis*	P42106
3.6.3.24	Nickel-transporting ATPase	*Escherichia coli*	P33593
2.1.1.143	24-methylenesterol C-methyltransferase	*Arabidopsis thaliana*	Q94JS4
2.1.1.143	24-methylenesterol C-methyltransferase	*Arabidopsis thaliana*	Q39227

All Swiss-Prot entries listed here have been updated with the corresponding EC number.

Several proposals have been made recently aimed at producing a complete catalog of biochemical activities, biological functions, and their cognate genes [2,3]. Many of these proposals recommend that such a project begin with prokaryotes because of the general ease of gene cloning from these species [1,2]. Indeed, our data support this notion, as we find substantially more orphans with a salvageability ranking of "good" and "excellent" in prokaryotes as compared to eukaryotes. The availability of a comprehensive review of the problem achieved by this survey, combined with broad genomic sequencing and powerful computational tools, leads us to conclude that the field is in an excellent position to rectify the information gap associated with the orphan activity phenomenon.

## Conclusion

More than one third of enzyme activities with assigned EC numbers are orphan activities, having no associated gene or protein sequence. We carried out a literature-based survey of a representative sample of presumed orphans intended to further validate and characterize these orphan activities. We have also assessed the practicability of identifying the genes associated with these orphans. In doing so, we captured data from the literature that should assist in future identification of cognate genes for the orphan activities we examined.

This survey confirmed that about 80% of sampled orphan activities have no sequence information associated with them, either in databases or in the literature. Weaknesses in database integration and failure to capture information from the literature account for most of the remaining 20%.

This survey points toward the significant scientific cost of having such a large fraction of characterized enzyme activities disconnected from sequence data. It also suggests that a larger effort, beginning with a comprehensive survey of all putative orphan activities, would resolve nearly 300 artifactual orphans and reconnect a wealth of enzyme research with modern genomics. For these reasons, we propose that a systematic effort to identify the cognate genes of orphan enzymes be undertaken.

## Methods

### Literature survey process

This survey was performed from June through August 2004 and relied on enzyme activities described by the NC-IUBMB. This enzyme classification and nomenclature system is hierarchical in nature and is based upon the reaction catalyzed. It assigns specific numerical identifiers, an EC number, to each distinct enzymatic activity. The first digit represents the class of reaction catalyzed (e.g., oxidoreductases are EC1; transferases are EC2). The second digit of the EC number refers to the subclass, which generally contains information about the type of compound or group involved (e.g., an enzyme acting on the CH-OH group of donors, or acting on the aldehyde or oxo group of donors). The third digit defines the sub-subclass, which specifies the nature of the reaction. The fourth digit is a serial number that is used to identify the individual enzyme within a sub-subclass (see [22] for a description of the classification system).

It is important to bear in mind that distinct proteins catalyzing the same reaction are assigned the same EC number. Since the EC system is based upon the reaction catalyzed, when applied to a protein it describes a biochemical function of this protein. That function can also be shared by several proteins (isozymes) that can be coded by genes in the same or different species.

Presumed orphan EC numbers were identified using the BioWarehouse database system [23]. BioWarehouse [24] is an integrated database that enables cross-database queries using the structured query language (SQL). SRI's BioWarehouse instance was queried for enzymatic activities with no matching sequences in any major protein sequence databases, including TrEMBL, PIR, SWISS-PROT, CMR, ENZYME, and BioCyc (the selection of these databases is described in [3]). This query returned an initial list of 1,356 EC numbers that had not been retired or merged at the time of the survey.

This list was randomized and the primary literature associated with a sample of these putative orphans was processed successively according to that random order. The size of the sample necessary to ensure representational accuracy as compared to the total pool of EC numbers was calculated using Equation 1. Approximately 180 orphans are required to achieve better than 95% confidence, given the total number of EC numbers. Since a sample of 228 orphans was ultimately surveyed, the 95% level of significance was exceeded.

#### Equation 1: sample size estimation

*SE *is the standard error associated with the survey, and is derived by dividing the sampling error by 1.96, such that for a sampling error of 5% (95% confidence interval), the standard error is 0.0255102. *p *is the probability that the EC number is a true positive, that is, there is truly no sequence information for that EC number; this value is 0.85 based on data from a preliminary survey. *N *is the universe of orphan activities. Solving for *n *provides the sample size.

SE^2 ^= [(p(1-p)/n)] [(N-n)/N]

A comprehensive manual analysis of the literature associated with this sample of 228 orphans drawn from the randomized list was performed as outlined in Figure 2. Various databases (Table 13) were consulted to extract the data elements listed in Table 12. For each selected putative orphan in the sample, the text search engine ExPASy Proteomics Server [25] was used to search TrEMBL, ENZYME, and IUBMB database records to confirm the absence of sequence data. For each orphan, all protein names, author names, reaction names, substrate names, and product names listed in the IUBMB record for that orphan were used as query arguments.

**Table 13:** Main data sources used by the orphan survey

**Database name**	**Content**	**Source**	**Accessed via...**
TrEMBL [32]	Comprehensive protein and DNA sequence data	Swiss Institute of Bioinformatics	Web
Comprehensive Microbial Repository (CMR [33])	Extensive genomic data for microbial species	The Institute for Genomic Research	BioWarehouse
BioCyc databases	Collection of pathway/genome databases primarily concerned with microbial species	Bioinformatics Research Group, SRI International	BioWarehouse
IUBMB Enzyme Nomenclature [34]	Description of enzymes that have been assigned an EC number by the Enzyme Commission	Nomenclature Committee of the International Union of Biochemistry and Molecular Biology	Web and BioWarehouse
ENZYME [35]	Repository of information relative to the nomenclature of enzymes	Swiss Institute of Bioinformatics	Web and BioWarehouse
NCBI Taxonomy [36]	Taxonomy database	National Center for Biotechnology Information	Web and BioWarehouse
PubMed	Literature database	National Library of Medicine	Web

The primary literature associated with each orphan's entry in the IUBMB database [5,26] describing the isolation and characterization of the activity was reviewed for the presence of sequence information. In particular, we were alert for the presence of molecular descriptors that might be useful in cloning the associated genes in the papers (described below), particularly M_r_, pI, and details of the purification scheme (Table 14). Systematic searches of PubMed were also performed to ascertain whether publications other than those cited by IUBMB might contain relevant sequence and molecular descriptor data. A total of 331 publications (1.45 papers per orphan) were examined for additional molecular descriptors that might be useful in cloning, as described above. Data obtained from these publications were assembled into a database.

**Table 14:** 

Name of enzyme activity
Is lack of sequence confirmed?
Bibliographical data (publication dates, authors, institutions)
Name of species
Can the species associated with the original publications be unambiguously identified?
Is a comprehensive genome sequence available for those species?
Are comprehensive genome sequences available from closely-related species?
Is there ongoing genomic sequencing for those species or from closely-related species?
Are molecular data such as M_r _and pI available?
Does the purification and characterization procedure suggest that purifying this enzyme should be reasonably straightforward?

All artifactual orphans (orphans for which sequence information was found during the literature review process) were reported promptly to the Swiss Institute of Bioinformatics, the European Bioinformatics Institute, the ORENZA database, and the Nomenclature Committee of the IUBMB for the relevant database entries to be updated.

### Data sources and database analyses

The initial searches for presumed orphan activities were performed using BioWarehouse version 3.0 (SRI International) running under Oracle 10G (Oracle Corporation, Redwood Shores, California). BioWarehouse is a bioinformatics data warehousing environment developed under the Bio-SPICE program [23].

### Identification and ranking of salvageable orphans

Salvageable orphans are orphan activities for which it is likely that at least one cognate gene can be identified and confirmed in a practical manner. The extent of this salvageability was determined by ranking validated orphans according to the likelihood and practicality that at least one cognate gene can be identified, and that the gene product can be isolated and demonstrated to catalyze the enzymatic activity in a practical manner.

Orphans were ranked based on data in the original literature, combined with the availability of the complete genome sequences for the species in which an orphan was first elucidated. The principal ranking factors are (1) clear identification of the species involved and its ease of growth; (2) the availability of molecular descriptors, most importantly the molecular mass (M_r_), but also the isoelectric point (pI); (3) the types of purification and analytical techniques used in the original literature; and (4) evidence that the protein can be purified with reasonable effort using current techniques, based on factors such as specific activity, purification yield, number of steps involved, and availability of substrate and of alternate purification procedures. "Excellent" and "Good" ratings indicate an activity associated with a sequenced organism, and whose purifications and assays are likely to be straightforward to replicate. "Difficult" activities are those with tricky purifications or complex assays, but a sequenced target organism or sequenced related organism. "Marginal" activities are those for which sequencing is in progress in the target organism, or a related organism. "Poor" activities are those for which no genome sequence is available, or sequencing is in progress in a related organism, and assay or purification conditions are likely to be hard to replicate.

#### Data availability

Information about validated orphan activities has been entered into the MetaCyc database [27]. Other data generated by our survey can be found at [9].

## Abbreviations

Enzyme Commission (EC), Nomenclature Committee of the International Union of Biochemistry and Molecular Biology (NC-IUBMB), Structured Query Language (SQL)

## Authors' contributions

PK conceived the study. PK and YP jointly devised the methodology. YP performed the literature research and drafted the manuscript. PK revised the manuscript.
